# Capacity of Ugandan public sector health facilities to prevent and control non-communicable diseases: an assessment based upon WHO-PEN standards

**DOI:** 10.1186/s12913-018-3426-x

**Published:** 2018-08-06

**Authors:** Hilary E. Rogers, Ann R. Akiteng, Gerald Mutungi, Adrienne S. Ettinger, Jeremy I. Schwartz

**Affiliations:** 1 The Heller School for Social Policy and Management at Brandeis University, Tufts University School of Medicine, Boston, USA; 2 Uganda Initiative for Integrated Management of Non-Communiable Diseases, Kampala, Uganda; 3 Programme for the Prevention and Control of Non-Communicable Diseases, Department of Community Health, Government of Uganda Ministry of Health, Kampala, Uganda; 4 Department of Nutritional Sciences, University of Michigan School of Public Health, Ann Arbor, MI USA; 5 Section of General Internal Medicine, Department of Medicine, Yale School of Medicine, New Haven, CT USA

**Keywords:** Uganda, Non-communicable disease, Prevention, Health facility readiness, Health service delivery

## Abstract

**Background:**

Non-communicable diseases (NCDs) are increasing in prevalence in low-income countries including Uganda. The Uganda Ministry of Health has prioritized NCD prevention, early diagnosis, and management. However, research on the capacity of public sector health facilities to address NCDs is limited.

**Methods:**

We developed a survey guided by the literature and the standards of the World Health Organization Pacakage of Essential Noncommunicable Disease Interventions for Primary Health Care in Low-Resource Settings. We used this tool to conduct a needs assessment in 53 higher-level public sector facilities throughout Uganda, including all Regional Referral Hospitals (RRH) and a purposive sample of General Hospitals (GH) and Health Centre IVs (HCIV), to: (1) assess their capacity to detect and manage NCDs; (2) describe provider knowledge and practices regarding the management of NCDs; and (3) identify areas in need of focused improvement. We collected data on human resources, equipment, NCD screening and management, medicines, and laboratory tests. Descriptive statistics were used to summarize our findings.

**Results:**

We identified significant resource gaps at all sampled facilities. All facilities reported deficiencies in NCD screening and management services. Less than half of all RRH and GH had an automated blood pressure machine. The only laboratory test uniformly available at all surveyed facilities was random blood glucose. Sub-specialty NCD clinics were available in some facilities with the most common type being a diabetes clinic present at eleven (85%) RRHs. These facilities offered enhanced services to patients with diabetes. Surveyed facilities had limited use of NCD patient registries and NCD management guidelines. Most facilities (46% RRH, 23% GH, 7% HCIV) did not track patients with NCDs by using registries and only 4 (31%) RRHs, 4 (15%) GHs, and 1 (7%) HCIVs had access to diabetes management guidelines.

**Conclusions:**

Despite inter-facility variability, none of the facilities in our study met the WHO-PEN standards for essential tools and medicines to implement effective NCD interventions. In Uganda, improvements in the allocation of human resources and essential medicines and technologies, coupled with uptake in the use of quality assurance modalities are desperately needed in order to adequately address the rapidly growing NCD burden.

**Electronic supplementary material:**

The online version of this article (10.1186/s12913-018-3426-x) contains supplementary material, which is available to authorized users.

## Background

Rapid population growth, urbanization, a nutritional transition, and indoor and outdoor air pollution are among the factors contributing to the growing burden of the major global non-communicable diseases (NCDs) in Uganda [1]. Based on a recent nationwide representative survey, the prevalence of hypertension, at 24.3%, approaches that in the United States [2]. The prevalence of diabetes mellitus is relatively low in urban (2.7%) and in rural (1%) areas, although it is expected to rise significantly in the near future [3]. Chronic obstructive pulmonary disease (COPD) in rural Uganda, where the prevalence is 16.2%, appears to be more common than in high-income countries [4]. The epidemiology of cancers is shifting from a predominance of HIV-associated malignancies to those more typically associated with “Western lifestyles” [5].

Despite this burden of risk factors and associated disease, as well as extensive experience in addressing chronic HIV infection, the Ugandan healthcare system remains ill-prepared to address NCDs [6]. Only a few prior studies have evaluated the capacity of public sector health facilties to provide NCD services. The Service Availability and Readiness Assessment (SARA) is a World Health Organization (WHO) -endorsed cross-sectional survey that provides insight into overall health facility readiness, though is limited in its acquisition of data specific to NCDs. To date, three SARA surveys have been conducted in Uganda: 2012, 2013, and 2014 with the latter being restricted to a sample of higher level facilties and conducted after the present study was completed [7]. The 2013 Uganda SARA survey sampled 209 public and private health facilities [8]. In that survey, the average availability of essential medicines to treat NCDs, across all facilities, was only 15% and regression analyses identified significant associations between availability of these medicines and region, managing authority, facility type, and HIV services [9]. Also, the tools necessary for the diagnosis and/or management of diabetes and cardiovascular disease were available in only 34 and 44% of facilities, respectively. One geographically limited study of overall readiness to manage chronic disease patients in the outpatient units of urban and rural health facilities revealed generally poor readiness to do so [10].

In 2006, in an effort to address the growing NCD burden in the country, the Ugandan Ministry of Health (MOH) created the Programme for the Prevention and Control of NCDs. In a collaboration between that Programme and the Uganda Initiative for Integrated Management of Non-Communicable Diseases, we conducted, in 2013, the first nationwide survey of public sector Ugandan health facilities focused specifically on readiness to provide services for NCDs. By doing so, we were able to conduct a more detailed assessment of NCD services than SARA surveys, which are intended to assess a wide-ranging spectrum of health service delivery readiness. We based our assessment of readiness on the standards set forth by WHO Package of Essential Noncommunicable Disease Interventions for Primary Health Care in Low-Resource Settings (WHO-PEN) [11].

## Methods

### Development of the needs assessment tool

We reviewed the published and grey literature to identify survey tools used in NCD-focused assessments conducted in other LMIC [11, 12]. Two of this study’s authors (HR and ARA) selected and adapted questions to include based on their relevance to Uganda’s hospital and facility structure and the MOH protocol for prevention and treatment of NCDs. The specific diseases of interest for this study were guided by official MOH priorities and included: diabetes, cancer, cardiovascular disease, chronic kidney disease, sickle cell disease, and COPD [13]. The medicines, technologies, and tools included in our survey are based upon the standards of WHO-PEN (Tables 1 and 2) [11]. Additional medicines, technologies, tools, and guidelines deemed essential by MOH were also included. The needs assessment tool was written collaboratively and was reviewed during a full-day meeting held in Kampala in 2013 and attended by 30 stakeholder groups including: MOH, Mulago National Referral Hospital, Regional Referral Hospitals (RRH), WHO-Uganda, and Uganda NCD Alliance (Appendix).

**Table 1:** WHO essential technologies and tools for implementing essential NCD interventions in primary care [11]

Technologies:	Tools:
Thermometer Stethoscope Blood pressure measurement device Measurement tape Weighing machine Peak flow meter Spacers for inhalers Glucometer Blood glucose test strips Urine protein test strips Urine ketones test strips	WHO/ISH risk prediction charts Evidence based clinical protocols Flow charts with referral criteria Patient clinical record Medical information register Audit tools
Add when resources permit	
Nebulizer Pulse oximeter Blood cholesterol assay Lipid profile Serum creatinine assay Troponin test strips Urine microalbuminuria test strips Tuning fork Electrocardiograph (if training to read and interpret electrocardiograms is available) Defibrillator

**Table 2:** WHO core list of medicines required for implementing essential NCD interventions in primary care [11]

NCD Medicines	
Thiazide diuretic Calcium channel blocker (amlodipine) Beta-blocker (atenolol) Angiotensin inhibitor (enalapril) Statin (simvastatin) Insulin Metformin Glibenclamide Isosorbide dinitrate Glyceryl trinitrate Furosemide Spironolactone Salbutamol Prednisolone Beclometasone Aspirin Paracetamol	Ibuprofen Codeine Morphine Penicillin Erythromycin Amoxcillin Hydrocortisone Epinephrine Heparin Diazepam Magnesium sulphate Promethazine Senna Dextrose infusion Glucose injectable solution Sodium chloride infusion Oxygen

### Sampling

Fifty-three public sector health facilities, including 14 Health Center IVs (HCIV), 26 General (District) Hospitals (GH), and 13 Regional Referral Hospitals (RRH), were surveyed (Appendix). These facility tiers were chosen because they were expected to offer NCD services [13]. Characteristics of each facility level have been previously published [10]. In 2012, in the Ugandan public sector, there were 13 RRH, 116 GH, and 170 HCIV [14].

All RRHs were surveyed. Head administrators of each RRH were asked to purposively select three GH or HCIV within their catchment areas (38 facilities). The selection criteria given to RRH staff included: equitable geographical location within the RRH region; basic NCD infrastructure already in place (clinics, equipment, and/or services); and availability of some human resources and basic infrastructure to implement service improvement activities. Given that the survey was funded to support a total of 53 facilities, the final two facilities were chosen randomly, for a total of 40 GH and HCIV.

### Data collection

Our study team, comprised of two authors (HR and ARA) and two additional MOH employees, conducted each assessment between July and November 2013. The survey tool was divided into 13 sections. Up to three members of the study team visited each facility and used the survey questionnaire to interview personnel from the facility department most relevant for each survey section. One team member interviewed the appropriate personnel while another team member manually entered the intereviewee’s responses into the survey. At larger facilities, each team member simultaneously interviewed personnel of different departments. These departments included: the Director’s office, facility administration, pharmacy, laboratory, outpatient clinics, and inpatient wards. Spot-checking of equipment or medicines was not formally incorporated into the survey.

Fifty-one surveys were completed by the study team and facility staff during these visits. Weather and road challenges prohibited the study team from visiting two facilities (Kitgum and Bududa GH). For these, surveys were completed by facility staff and sent back to MOH. Gaps and inconsistencies were clarified, prior to analysis, by way of follow-up calls to the relevant contacts in the facilities. Despite these efforts, the response rate for some survey variables was less than 100%. The denominators listed throughout the Results section of this paper indicate the total number of facilities for which we have data for each variable.

### Analysis

All survey data were entered into a Microsoft Access 2013 database and all data analyses were conducted using SAS (version 9.2). Data were analyzed according to the organization of the original questionnaire (Additional file 1). Descriptive statistics (N, % and Mean ± SD, where applicable) were calculated for each of the major categories of services, equipment, pharmaceuticals, and laboratory services for each facility type (RRH, GH, and HCIV). Such analyses were deemed by MOH to be the most helpful for identifying gaps and providing insight into where MOH and funders’ resources are most needed across the RRHs, GHs, and HCIVs.

### Dissemination

The results of this needs assessment were compiled as a Report and shared with the MOH NCD Unit in order to guide the procurement and distribution of additional health supplies.

## Results

### Description of sample

Thirteen RRH, 26 GH, and 14 HCIV were surveyed. Eight of 9 (88.9%) RRH, 6/21 (28.6%) GH, and 3/12 (25%) HCIVs self-classified as urban (Table 3).

**Table 3:** Health facilities surveyed, by geographic category

Type of Facility	Total Surveyed	Geographic category	
		Urban n/Total respondents (%)	Rural n/Total respondents (%)
Regional Referral Hospital (RRH)	13	8/9 (88.9)	1/9 (11.1)
General Hospital (GH)	26	6/21 (28.6)	15/21 (71.4)
Health Center IV (HCIV)	14	3/12 (25)	9/12 (75)

### Health personnel

We have previously reported degrees of self-reported confidence in NCD management among staff of various cadres at the sampled facilities, as derived from other sections of this survey [1]. In addition to those data, we recorded the numbers of healthcare personnel relevant to the care of patients with NCDs who were working at each facility. As expected, since we only sampled higher level facilities, the most common cadre of medical provider at all levels of facility sampled were Medical Officers (doctors who have completed an internship) and Clinical Officers (non-medical doctor clinicians). Specialist physicians and specialist nurses were rare at the sampled facilities, with 6 (46.2%) RRH reporting at least one radiologist but only 1 (7.7%) reporting a cardiologist or an endocrinologist. Few facilities reported having access to other cadres of health professionals important in the ongoing care of patients with NCDs, such as nutritionists, diabetes educators, who were on staff at 1 (7.7%) RRH and 1 (3.8%) GH, or foot care specialists (Table 4).

**Table 4:** Health care personnel staffing, by health facility level

Personnel Type	Regional Referral Hospitals (*N* = 13)		General Hospitals (*N* = 26)		Health Center IVs (*N* = 14)	
	No. reporting at least 1 staff member of this cadre n (%)	Mean no. of members (±SD)	No. reporting at least 1 staff member of this cadre n (%)	Mean no. of members (±SD)	No. reporting at least 1 staff member of this cadre n (%)	Mean no. of members (±SD)
General Practitioners						
Family physician	3 (23.1)	1.7 (± 1.2)	3 (11.5)	1.0 (± 0.0)	0 (0.0)	–
Medical officer	10 (76.9)	5.3 (± 4.3)	23 (88.4)	3.4 (±1.9)	11 (78.6)	1.2 (±0.7)
Clinical officer	13 (100.0)	10.6 (± 3.9)	23 (88.4)	6.0 (±3.1)	11 (78.6)	1.2 (±1.3)
Specialist Physicians						
Cardiologist	1 (7.7)	1.0 (± 0.0)	1 (3.8)	5.0 (± 0.0)	3 (21.4)	1.7 (±0.6)
Endocrinologist/diabetologist	1 (7.7)	1.0 (± 0.0)	1 (3.8)	2.0 (± 0.0)	0 (0.0)	–
Neurologist	0 (0.0)	–	1 (3.8)	1.0 (± 0.0)	0 (0.0)	–
Oncologist	0 (0.0)	–	0 (0.0)	–	0 (0.0)	–
Pathologist	1 (7.7)	1.0 (± 0.0)	0 (0.0)	–	0 (0.0)	–
Psychiatrist	1 (7.7)	1.0 (± 0.0)	2 (7.7)	1.0 (± 0.0)	3 (21.4)	1.0 (± 0.0)
Pulmonologist	0 (0.0)	–	0 (0.0)	–	0 (0.0)	–
Radiologist	6 (46.2)	1.8 (± 1.3)	0 (0.0)	–	0 (0.0)	–
Nurse						
Nurse - general	10 (76.9)	47.8 (± 22.2)	22 (84.6)	33.2 (±15.8)	13 (92.9)	5.4 (±1.7)
Nurse - diabetic	4 (30.8)	3.0 (± 1.0)	7 (26.9)	1.4 (±0.8)	2 (14.3)	2.5 (±2.1)
Nurse - psychiatric	9 (69.2)	3.9 (± 3.3)	20 (76.9)	1.6 (±0.9)	8 (57.1)	1.8 (±0.5)
Other						
Community health worker	7 (53.8)	2.6 (± 0.9)	7 (26.9)	5.3 (±6.6)	2 (14.3)	1.9 (± 2.1)
NCD Counselor	0 (0.0)	–	1 (3.8)	2.0 (± 0.0)	0 (0.0)	–
Foot care specialist	1 (7.7)	1.0 (± 0.0)	0 (0.0)	–	0 (0.0)	–
Social worker	6 (46.2)	1.6 (± 0.9)	11 (42.3)	1.1 (±0.3)	0 (0.0)	–
Diabetes educator	1 (7.7)	1.0 (± 0.0)	1 (3.8)	6.0 (± 0.0)	0 (0.0)	–
Nutritionist	8 (61.5)	1.3 (± 0.5)	8 (30.8)	1.1 (± 0.35)	0 (0.0)	–

### Essential services and technologies

None of the sampled facilities reported having all of the essential medical devices recommended by WHO-PEN (Table 1). Less than half of all RRHs and GHs had at least one of each type of blood pressure machine (mercury sphygmomanometer, aneroid, an/or automated). For facilities with non-physician health workers, WHO recommends a blood pressure measurement device with digital reading. Five out of the 14 HCIVs (35.7%) had at least one automated blood pressure machine. Six (42.9%) had at least one aneroid machine and one mercury sphygmomanometer. Few facilities reported having blood pressure cuffs: 4 (30.8%) RRHs, 9 (34.6%) GHs, and 8 (57.1%) HCIVs reported having at least one standard adult cuff in the facility and far less reported having a pediatric cuff. Half of all types of health facilities (7 (53.8%) RRHs, 13 (50%) GHs, and 7 (50%) HCIVs) had at least one measuring tape which is used for anthropometric measurements such as height and waist circumference. Of the facilities surveyed, 8 (61.5%) RRHs, 19 (73.1%) GHs, and 6 (42.9%) HCIVs had at least one glucometer. Three RRHs (23.1%), 2 GHs (7.7%), and 2 HCIVs (14.3%) had urine testing strips for protein and ketones (Table 5).

**Table 5:** Availability of basic clinical equipment, by health facility level

Equipment	No. of facilities (by type) that have at least one of equipment		
	RRH (N = 13) n (%)	GH (N = 26) n (%)	HCIV (N = 14) n (%)
Thermometer	7 (53.8)	14 (53.9)	8 (57.1)
Stethoscope	8 (61.5)	16 (61.5)	7 (50.0)
Blood pressure machine: Mercury sphygmomanometer	6 (46.2)	9 (34.6)	6 (42.9)
Blood pressure machine: Aneroid	6 (46.2)	7 (26.9)	6 (42.9)
Blood pressure machine: Automated	5 (38.5)	13 (50.0)	5 (35.7)
BP cuffs: Standard (25 cm × 12 cm)	4 (30.8)	9 (34.6)	8 (57.1)
BP cuffs: Alternate (36 cm × 12 cm)	0 (0.0)	1 (3.8)	1 (7.1)
BP cuffs: Pediatric	1 (7.7)	3 (11.5)	3 (21.4)
Measuring tapes	7 (53.8)	13 (50.0)	7 (50.0)
Weighing scales: Bathroom type	7 (53.8)	15 (57.7)	8 (57.1)
Weighing scales: Hospital type	4 (30.8)	11 (42.3)	4 (28.6)
Glucometer	8 (61.5)	19 (73.1)	6 (42.9)
Urine testing strips	3 (23.1)	2 (7.7)	2 (14.3)
Monofilament	1 (7.7)	1 (3.8)	0 (0.0)
Spirometer	1 (7.7)	0 (0.0)	0 (0.0)
Spacers for inhalers	3 (23.1)	1 (3.8)	1 (7.1)
Nebulizer	4 (30.8)	2 (7.7)	2 (14.3)
All of the above	0 (0.0)	0 (0.0)	0 (0.0)
None of the above	1 (7.7)	2 (7.7)	2 (14.3)

### Advanced technologies

We collected data on the availability of advanced clinical technologies, though these are not considered essential and, therefore, are not included in WHO-PEN. More than half of each type of health facility had an ultrasound scan, but not all of them were functional at the time of the survey. For example, 11 (84.6%) RRHs had an ultrasound scan, but only 6 (54.6%) of those were functional. Three (23.1%) RRHs had an electrocardiogram (ECG) machine though only 1 (33.3%) of these was functional. Ten (76.9%) RRHs and 21 (80.8%) GHs had X-ray machines, but just over half of these were functional (6; 60.0% and 11; 52.4%, respectively). Two (14.3%) HCIVs had X-ray machines, but neither of them were functional. Even fewer health facilities had other types of advanced equipment. For example, 38.5% of RRHs had a Doppler machine and 15.4% had a CT-scan. Few health facilities reported having a reliable power supply necessary to power these advanced pieces of clinical equipment. Specifically, 53.9% of RRHs, 61.5% of GHs, and 28.6% of HCIVs reported having a reliable power supply. Although not always functional, 69.2% of RRHs, 73.1% of GHs, and 64.3% of HCIVs had an alternative source of power which was usually a generator or solar panels (Table 6).

**Table 6:** Availability and function of advanced clinical equipment, by health facility level

Equipment	Regional Referral Hospitals (N = 13)		General Hospitals (N = 26)		Health Center IVs (*N* = 14)	
	Available n (%)	Functional n (%)	Available n (%)	Functional n (%)	Available n (%)	Functional n (%)
Ultrasound Scan	11 (84.6)	6 (54.6)	22 (84.6)	17 (77.3)	7 (50.0)	3 (42.9)
ECG Machine	3 (23.1)	1 (33.3)	4 (15.4)	2 (50.0)	1 (7.2)	0 (0.0)
X-Ray	10 (76.9)	6 (60.0)	21 (80.8)	11 (52.4)	2 (14.3)	0 (0.0)
CT-Scan	2 (15.4)	1 (50.0)	0 (0.0)	0 (0.0)	0 (0.0)	0 (0.0)
Doppler	5 (38.5)	3 (60.0)	1 (3.8)	0 (0.0)	0 (0.0)	0 (0.0)
Reliable power supply	7 (53.8)	4 (57.1)	16 (61.5)	13 (81.3)	4 (28.6)	2 (50.0)
Alternative source of power	9 (69.2)	7 (77.8)	19 (73.1)	17 (89.5)	9 (64.3)	4 (44.4)
All of the above	1 (7.7)	1 (7.7)	0 (0.0)	0 (0.0)	0 (0.0)	0 (0.0)
None of the above	2 (15.4)	4 (30.8)	2 (7.7)	5 (19.2)	3 (21.4)	8 (57.1)

### Laboratory services

All of the RRHs and GHs (100%) and 12 of the HCIVs (86%) had an on-site laboratory. In general, RRHs had the highest proportion of facilities whose laboratories offered general tests, such as hemoglobin, complete blood count and differential, electrolytes, renal function tests, liver function tests, lipid profile, and urinalysis. The only laboratory test uniformly available at all surveyed facilities was random blood glucose. The ability to perform a HbA1c, an important test for diabetes monitoring, was limited to five (19.2%) GHs and was unavailable in RRHs. Hemoglobin electrophoresis, a fairly sophisticated test that can be used for diagnosing sickle cell disease, was also limited to GHs and unavailable in RRHs. The availability of additional laboratory services are shown in Table 7.

**Table 7:** Laboratory equipment and test availability, by health facility level

On-site laboratory	Number of facilities with laboratory equipment or test		
	RRH (N = 13) n (%)	GH (N = 26) n (%)	HCIV (N = 14) n (%)
	13 (100%)	26 (100%)	12 (86%)
General tests			
Hemoglobin	12 (92.3)	23 (88.5)	11 (78.6)
Complete blood count and differential	11 (84.6)	19 (73.1)	5 (35.7)
Electrolytes	9 (69.2)	10 (38.5)	0 (0.0)
Renal function	9 (69.2)	13 (50.0)	1 (7.1)
Liver function	9 (69.2)	11 (42.3)	0 (0.0)
Lipid profile	6 (46.2)	8 (30.8)	1 (7.1)
Urinalysis	11 (84.6)	25 (96.2)	13 (92.9)
All of the above	6 (46.2)	6 (23.1)	0 (0.0)
None of the above	1 (7.7)	0 (0.0)	0 (0.0)
Diabetes tests			
Random blood glucose	12 (92.3)	23 (88.5)	14 (100.0)
Hemoglobin A1c	0 (0.0)	5 (19.2)	0 (0.0)
Urine microalbumin	4 (30.8)	3 (11.5)	0 (0.0)
All of the above	0 (0.0)	1 (3.8)	0 (0.0)
None of the above	1 (7.7)	2 (7.7)	0 (0.0)
Cancer tests			
Cytology	2 (15.4)	3 (11.5)	0 (0.0)
Fecal occult blood	3 (23.1)	4 (15.4)	0 (0.0)
Prostate-Specific Antigen	1 (7.7)	2 (7.7)	1 (7.1)
All of the above	0 (0.0)	0 (0.0)	0 (0.0)
None of the above	8 (61.5)	19 (73.1)	13 (92.9)
Sickle cell disease tests			
Hemoglobin electrophoresis	0 (0.0)	4 (15.4)	0 (0.0)
Sickling test	11 (84.6)	21 (80.8)	7 (50.0)
All of the above	0 (0.0)	2 (7.7)	0 (0.0)
None of the above	2 (15.4)	4 (15.4)	7 (50.0)

### Essential medicines for NCDs

The standard protocol for assessing availability of essential medicines, according to SARA, is a visual spot-check of dispensary shelves which we did not conduct. However, given that National Medical Stores (NMS) resupplies facilities with medicines every two months [15], resulting in variability in medicine stock over time, we attempted to gather information regarding trends in medicine availability. Eleven (69%) RRHs, 15 GHs (58%), and 9 HCIVs (64%) reported they routinely did not receive the drugs requested from NMS. Ten (77%) RRHs, 17 (65%) GHs, and 10 HCIVs (71%) reported they routinely did not receive the requested quantity of particular drugs. All facilities surveyed reported experiencing stock-outs of at least one class of essential medicine for NCDs within the previous year. Availability and stock-outs of medicines used to treat hypertension and diabetes are shown in Table 8.

**Table 8:** Availability of medicines for hypertension and diabetes, by health facility level

Pharmaceutical Classes		RRH (N = 13)			GH (N = 26)			HCIV (N = 14)		
		No. that have drug available	Stockout in last quarter	Stockout in last year	No. that have drug available	Stockout in last quarter	Stockout in last year	No. that have drug available	Stockout in last quarter	Stockout in last year
		n (%)	n (%)	n (%)	n (%)	n (%)	n (%)	n (%)	n (%)	n (%)
Hypertension										
Thiazide diuretic		12 (92.3)	1 (7.7)	2 (15.4)	23 (88.5)	3 (11.5)	2 (7.7)	9 (64.3)	3 (21.4)	2 (14.3)
Calcium channel blocker		12 (92.3)	1 (7.7)	1 (7.7)	22 (84.6)	2 (7.7)	3 (11.5)	14 (100.0)	4 (28.6)	5 (35.7)
Beta-blocker		7 (53.8)	3 (23.1)	3 (23.1)	14 (53.8)	9 (34.6)	7 (26.9)	13 (92.9)	3 (21.4)	3 (21.4)
ACE inhibitor		10 (76.9)	2 (15.4)	2 (15.4)	20 (76.9)	5 (19.2)	6 (23.1)	6 (42.9)	2 (14.3)	2 (14.3)
Others (e.g. Methyldopa, hydralazine, magnesium sulphate)		10 (76.9)	2 (15.4)	2 (15.4)	20 (76.9)	2 (7.7)	5 (19.2)	9 (64.3)	3 (21.4)	2 (14.3)
Diabetes										
Biguanides		12 (92.3)	0 (0.0)	0 (0.0)	23 (88.5)	4 (15.4)	2 (7.7)	14 (100.0)	4 (28.6)	5 (35.7)
Sulfonylureas		11 (84.6)	2 (15.4)	2 (15.4)	21 (80.8)	6 (23.1)	2 (7.7)	11 (78.6)	3 (21.4)	4 (28.6)
Thiazolidinediones		1 (7.7)	2 (15.4)	2 (15.4)	2 (7.7)	5 (19.2)	4 (15.4)	1 (7.1)	1 (7.1)	1 (7.1)
Dipeptidyl peptidase-4 inhibitors			1 (7.7)	1 (7.7)	1 (3.8)	5 (19.2)	4 (15.4)	1 (7.1)	1 (7.1)	0 (0.0)
Alpha-glucosidase inhibitors			1 (7.7)	1 (7.7)	2 (7.7)	5 (19.2)	5 (19.2)	0 (0.0)	0 (0.0)	1 (7.1)
Insulin type	Ultra short-acting	8 (61.5)	1 (7.7)	0 (0.0)	9 (34.6)	3 (11.5)	6 (23.1)	3 (21.4)	0 (0.0)	0 (0.0)
	Short-acting	9 (69.2)	3 (23.1)	3 (23.1)	14 (53.8)	2 (7.7)	6 (23.1)	5 (35.7)	1 (7.1)	1 (7.1)
	Intermediate	10 (76.9)	3 (23.1)	5 (38.5)	12 (46.2)	4 (15.4)	6 (23.1)	3 (21.4)	0 (0.0)	0 (0.0)
	Long-acting	11 (84.6)	3 (23.1)	4 (30.8)	14 (53.8)	3 (11.5)	5 (19.2)	3 (21.4)	1 (7.1)	1 (7.1)

### Specialty NCD clinics

The most common type of subspecialty clinic type at the sampled health facilities was a Diabetes Clinic. These were available in 11 RRHs (84.6%) and 14 GHs (53.8%). Although specialty clinics are not expected in Ugandan HCIVs, 3/14 (21.4%) offered a Diabetes Clinic (Table 9).

**Table 9:** Sub-specialty NCD clinics, by health facility level

Clinics/Wards	RRH (N = 13) n (%)	GH (N = 26) n (%)	HCIV (N = 14) n (%)
Diabetes	11 (84.6)	14 (53.8)	3 (21.4)
Hypertension	7 (53.9)	5 (19.2)	1 (7.1)
Cancer	2 (15.4)	1 (3.8)	0 (0.0)
Cardiology	2 (15.4)	1 (3.8)	0 (0.0)
COPD	1 (7.7)	0 (0.0)	0 (0.0)
Renal	2 (15.4)	0 (0.0)	0 (0.0)
Sickle Cell	4 (30.8)	2 (7.7)	0 (0.0)
All of the above	0 (0.0)	0 (0.0)	0 (0.0)
None of the above	1 (7.7)	11 (42.3)	11 (78.6)

### Patient support and education

The range of activities offered at facilities were broad, varying from printed handouts to more structured in person support services. Individual NCD education - one-on-one encounters between a health professional and a patient to improve the patient’s understanding of chronic disease (s) as well to create a self-management plan - was offered at 9 (69.2%) RRHs and 17 (65.4%) GHs, while group education was offered at 12 (92.3%) RRHs and 18 (69.2%) GHs. Eight (61.5%) RRHs and 15 (57.7%) of GHs offered foot care for diabetic patients. Nutritional advice was offered to patients at most facilties, though only 4 (30.8%) RRHs, 2 (7.7%) GHs, and 2 (14.3%) of HCIVs provided NCD Information, Education and Communication (IEC) materials, such as posters and brochures. Ten (77.0%) RRHs offered self-management NCD support and provided linkages to peer/social group NCD support. Thirteen (50%) GHs and 2 (14.3%) HCIVs offered self-management NCD support and 10 (38.5%) and 3 (21.4%), respectively, provided linkages to peer/social group NCD support (Table 10).

**Table 10:** NCD education and support, by health facility level

Education or support service	RRH (N = 13) n (%)	GH (N = 26) n (%)	HCIV (N = 14) n (%)
Individual NCD education	9 (69.2)	17 (65.4)	5 (35.7)
Group NCD education	12 (92.3)	18 (69.2)	4 (28.6)
Foot care for diabetic patients	8 (61.5)	15 (57.7)	2 (14.3)
Nutrition advice for all patients	12 (92.3)	23 (88.5)	11 (78.6)
NCD Information, Education and Communication (IEC) materials	4 (30.8)	2 (7.7)	2 (14.3)
Self-management NCD support	10 (77.0)	13 (50.0)	2 (14.3)
Peer/social group NCD support	10 (77.0)	10 (38.5)	3 (21.4)
All of the above	2 (15.4)	2 (7.7)	0 (0.0)
None of the above	1 (7.7)	1 (3.8)	1 (7.1)

### Quality assurance

WHO-PEN considers the use of patient registries and management guidelines among the minimum quality assurance standards for NCD care at health facilities.**Error! Bookmark not defined.** Most surveyed facilities also did not track patients with NCDs by using registries. Only six (46.2%) RRHs were using such registries for new NCD cases in at least one type of NCD clinic. Only 4 (30.8%) RRHs, 4 (15.4%) GHs, and 1 (7.1%) HCIVs reported having access to diabetes management guidelines (Table 11). Even fewer reported having access to guidelines on management of hypertension, hyperlipidemia, and asthma, and screening and treatment of tobacco use, cancer, mental health, or sickle cell disease.

**Table 11:** Availability of NCD guidelines, by health facility level

Registries and Guidelines	RRHs N = 13 n (%)	GHs N = 26 n (%)	HCIVs N = 14 n (%)
NCD registry for new cases	6 (46.2)	6 (23.1)	1 (7.1)
Diabetes management	4 (30.8)	4 (15.4)	1 (7.1)
Hypertension management	3 (23.1)	3 (11.5)	1 (7.1)
Hyperlipidemia management	0 (0.0)	2 (7.7)	0 (0.0)
Tobacco screening & treatment	0 (0.0)	2 (7.7)	0 (0.0)
Alcohol screening & treatment	0 (0.0)	1 (3.9)	1 (7.1)
Cancer (cervical, breast, prostate) screening & treatment	3 (23.1)	2 (7.7)	2 (14.3)
Mental health screening & treatment	2 (15.4)	3 (11.5)	2 (14.3)
Asthma management	2 (15.4)	2 (7.7)	1 (7.1)
Sickle cell screening & management	2 (15.4)	3 (11.5)	0 (0.0)
Palliative care	2 (15.4)	6 (23.1)	1 (7.1)
All of the above	0 (0.0)	0 (0.0)	0 (0.0)
None of the above	4 (30.8)	13 (50.0)	10 (71.4)

### Procurement and distribution

In July 2015, these data were utilized by MOH to guide the distribution of newly acquired essential equipment to the 53 surveyed health facilities. The procurement and distribution included: HbA1c machines and supplies, glucometers and glucose strips, weighing scales, BP machines, tuning forks, reflex hammers, tape measures, stethoscopes, stadiometers, fundoscopes, and monofilaments.

## Discussion

Results of this assessment of the capacity of Ugandan health facilities to address NCDs demonstrate areas of strength as well as significant gaps in the availability of equipment, medicines, and laboratory tests. Although there is variability among the different types of health facilities, none of the facilities surveyed meet the WHO-PEN standards for essential tools and medicines to implement effective NCD interventions [10].

As expected, the RRHs largely fared better than GHs and HCIVs, but a concerning number of facilities lacked equipment, drugs, and standard guidelines for basic and effective NCD prevention and control, based on the WHO-PEN. Particularly concerning at these higher level facilities were the shortcomings in the supply of basic essential technologies. Providers working at health facilities lacking blood pressure machines, measuring tapes, and scales cannot effectively risk-stratify their patients for cardiovascular disease. Without glucose testing strips, providers cannot be expected to deliver adequate care for patients with diabetes.

In addition to basic technologies, all facilities experienced concerning numbers of essential NCD medicines stockouts within the previous year. The recent USAID-funded Securing Ugandans’ Right to Essential Medicines (SURE) [16] is an example of promising work focused on improving access to medicines in the country. However, despite efforts such as SURE, recent evidence demonstrates the continued shortages of, and disparities in, essential medicine availability in Uganda [9]. When medicines and basic technologies are unavailable in the public sector, patients must procure these for themselves in the private sector. However, despite generally better availability, access for patients is still highly restricted by cost. A recent study in Uganda found that 27% of medicines and 32% of diagnostic tests for diabetes and cardiovascular disease were affordable, based on a cost of less than or equal to three days’ wages of a low-level government worker [17]. The importance of improving the supply chain for NCD medicines deserves special mention particularly given that medicine resupply from NMS occurs only every two months. A reduction in stock-outs of NCD medicines will not be achievable without improvements in their supply chain networks. The distribution network for NCD medicines is much less refined than that of HIV medicines which are acquired and distributed though donor-funded mechanisms with sophisticated supply chains [18]. Lessons learned from the successes of HIV supply chains and can be applied to NCD supply chains with minimal investment [19] .

There are a number of concerning features regarding the presence of clinical equipment at the surveyed facilities including high rates of non-functional equipment. Ultrasound machines are often used in the diagnosis and management of chronic kidney disease that is a common outcome of diabetes and hypertension. X-rays are important tests in the management of lung diseases, both communicable (such as tuberculosis) and non-communicable (such as COPD).

The 53 higher level public sector Ugandan health facilities surveyed generally lacked basic NCD prevention and screening services. Though community-based prevention and screening activities are an important component for the promotion of healthy lifestyles and early detection of NCDs [20], health facilities play a vital role in the continuum of services for patients. Despite this, less than half of all health facilities have the essential equipment and tests to screen for NCDs. Many of the facilities do not conduct basic and necessary NCD services, especially at the GH and HCIV level. This is especially concerning given the primary health care focus of these facilities and the likelihood that these facilities see the same patients more regularly than RRHs and therefore have more opportunity to prevent, identify, and manage NCDs. One area of strength in our findings was the availability of individual and group NCD education and nutritional counseling, most of which were offered at a majority of sampled facilities. In addition, 77% of RRHs sampled offered peer NCD support groups. Effective NCD prevention efforts must be more robust and multi-faceted. These should include proven population-level strategies, such as the WHO Best Buys [21], public health awareness campaigs using mass/social media, and community empowerment efforts such as at schools and places of work [22]. But public health campaigns must also be accompanied by individual-level health promotion efforts. At the individual patient level, health promotion education can take many forms [23]. This includes Information, Education, and Communication (IEC) materials, which were offered at only 14.3% of HCIVs and 7.7% of GHs in our study but could also include innovative self-management tools such as illustrated booklets [24] and mobile health platforms that might be leveraged to include NCD-related educational content. Alcohol and tobacco screening was reported to occur in only a small fraction of sampled facilities but these are critical elements of NCD prevention that can easily be performed by healthcare providers and are proven effective [25, 26].

The health facilities in our study also had poor quality assurance methods in place for NCD services. Only 11.5 and 15.4% of GHs in our study utilized diabetes and hypertension guidelines, respectively. A similar study in Tanzania found that 13% of facilities used guidelines for these conditions [27]. Algorithmic approaches to chronic disease management are especially critical in lower-level facilities in which care is provided by non-medical-doctor clinicians. Integrated approaches to comprehensive NCD care, such as WHO HEARTS, which was developed based on WHO-PEN, must incorporate management algorithms that can be easily followed and are locally appropriate and rational for the setting.

This study also highlights the urgent need for tools to allow countries like Uganda to frequently measure and monitor the availability of health service delivery indicators. SARA, an extensive and robust WHO-endorsed survey tool, is meant to be conducted annually. However, the most recent SARA in Uganda was completed in 2014. Such infrequent assessments of health system readiness and service availability create major challenges to the proper planning and effective distribution of limited resources. Health systems face many competing priorities. Not all NCDs are of equal burden and, therefore, not all essential services can, nor should necessarily, be distributed equally. An example in Uganda would be hypertension, which has a prevalence of 26.4% [28] compared to diabetes which has a prevalence of 1.4% [3].This is the basis for WHO-PEN, which itself represents a core set of low-cost, high-impact NCD interventions that can be delivered through a primary care approach [29].

Given that there were three SARA surveys conducted in Uganda between 2012 and 2014, we feel it is important to point out a number of specific ways in which our study adds to the evidence available from those surveys. The 2014 SARA survey was the most similar to our survey in terms of health facilities sampled insofar as it was restricted to HCIV and above. This SARA survey, like our study, inquired about functionality of equipment during the deployment of the survey However, the NCD-related content of this survey was limited to the availability of a few basic pieces of equipment (ie blood pressure machine and stethoscope) and 20 essential medicines. The 2012 and 2013 SARA surveys sampled a wider array of facility types but also were more comprehensive in terms of NCD-related scope. Our assessment of service availability included the presence of many types of personnel, including nutritionists, social workers, and diabetes nurses, among others, and assessed confidence. Comprehensive NCD care requires a team-based approach and continuing education to build confidence and this is not assessed in SARA. SARA assesses medicine availability by spot-checking on pharmacy shelves, while we assessed reported trends in medicine availability over time by inquiring about stock-outs. While neither methodology adequately substitutes for real-time monitoring of availability, we feel these two techniques complement one another and could both be employed in future work. The 2013 SARA reported 80% availability of CVD guidelines, while we found markedly lower availability of these, as discussed earlier. This is illuminating and we feel is related to methodology. While SARA spot-checks for availability of guidelines at facilities, the fact that we asked clinical leaders about the presence of guidelines was likely more indicative of the likelihood that they are actually being used in clinical practice. NCD care requires tools, such as ECG, that are considered by WHO-PEN to be advanced technologies. SARA does not survey for these items. Not only was ECG available in a minority of sampled facilities, but in only 5.6% of all sampled facilities was ECG functional. Future nationally representative assessments of health facility capacity to address NCDs might be bolstered by incorporating some of the methodologies employed by our study.

The local dissemination of our findings led to a guided procurement and distribution process, as described above. Though the number of each of these items was modest, this represents an important appropriation of essential tools by MOH based on the findings of this survey. Documentation of system gaps should always be met by attempts to close those gaps. We believe that the alignment of this needs assessment with MOH priorities and the strategic dissemination process likely aided this process and should serve as a model to other researchers aiming to document health system deficiencies.

This study has a number of limitations. First, the study sample was not randomly selected and did not include all of the GHs or HCIVs. Consequently, the results from this needs assessment may not be fully representative. Second, the “gold standard” of conducting a needs assessment is time-intensive direct observation [12]. However, due to time and funding constraints, each survey was completed in less than one day and the quality and accuracy of the data collected depended on the staff available on the particular day of the study team visit. The levels of knowledge and experience of personnel who helped to complete the survey varied at each facility which may have affected the accuracy of reports. Therefore, future improvements for similar needs assessment surveys would include randomized sampling, standardized data collection methods [30], and time-intensive direct observation with manual spot checks. A more sustainable alternative, though one with substantial associated up-front costs, would be digitization of supply chain management which would allow for improved transparency and data access by individuals within facilities and in health sector planning positions. Finally, in some cases, we observed unexpectedly lower proportions of available essential medicines and technologies in RRHs than in GHs. In our experience, there were likely two explanations for this finding. First, some GHs receive donations from non-governmental organizations rendering them better equipped than their counterparts. Second, facilities differ in their organizational structures and administrative management. Those that are more organized and better managed are likely to be better equipped. Recent analyses of Ugandan SARA data also revealed that some GHs were better supplied than some RRHs [**Error! Bookmark not defined.**]. Despite these limitations, this needs assessment provided data and information about Ugandan health facilities that was previously unknown and can be considered complementary to other sources of facility readiness data.

## Conclusions

This first nationwide research study of the capacity of higher level public sector health facilities in Uganda to provide NCD services highlights many critical gaps. These gaps were addressed, to a limited extent, throught the resulting procurement and distribution process. However, deficiencies such as those of healthcare personnel quantity and training, and access to medicines will continue to limit the potential for improving the quality of NCD service delivery in the country. This study demonstrates the need for Uganda to scale-up low cost, high impact NCD interventions and strengthen the capacity of health personnel to reduce NCD-related disability and death. Improvement of the capacity of health facilities and personnel to effectively detect and manage NCDs can yield significant economic gain for Uganda through a reduction in premature mortality, improved quality of life, and increased productivity [11]. Finally, as the world prepares for the third United Nations General Assembly high-level meeting on NCDs later this year, it is imperative to state that any system changes that seek to fill gaps such as those identified in this study must come in the context of attention to equitable distribution of resources and attention to the socioecomonic determinants of health [21].

### Additional file

Additional file 1: MOH Needs Assessment Tool. Non-Communicable Diseases Needs Assessment Tool used in this study. (DOCX 93 kb)

## Appendix

**Table 12:** Description of participants in July 26, 2013 review meeting in Kampala, Uganda

Organization/Institution (n)	Description of participants (n)
Ministry of Health (6)	Technical staff from: Health promotion (1) Non-communicable disease program (5)
Mulago National Referral Hospital (7)	Physician consultants (5) Pediatric endocrinologist (1) Medical officer (1) Departments represented include cardiology, oncology, endocrinology, pulmonology, and general medicine
Regional referral hospitals (12)	Directors, who are also physicians and surgeons (5) Physicians (6) Medical officer (1)
World Health Organization-Uganda (1)	NCD officer (1)
Uganda NCD Alliance (3)	Physicians (2) Coordinator (1)

**Table 13:** List of health facilities surveyed

Regional Referral Hospitals (n = 13)	General Hospitals (n = 26)	Health Center IVs (n = 14)
Arua RRH	Apac Hospital	Alebtong HCIV
Fort Portal RRH	Kaabong Hospital	Amuria HCIV
Gulu RRH	Tororo Hospital	Budaka HCIV
Hoima RRH	Anaka Hospital	Dokolo HCIV
Jinja RRH	Fort Portal RRH	Kabwohe HCIV
Kabale RRH	Kisoro Hospital	Kamukira HCIV
Lira RRH	Lyantonde Hospital	Kassanda HCIV
Masaka RRH	Bundibugyo Hospital	Kebisoni HCIV
Mbale RRH	Itojo Hospital	Koboko HCIV
Mbarara RRH	Rakai Hospital	Nabilatuk HCIV
Moroto RRH	Kyenjojo General Hosp.	Padibe HCIV
Mubende RRH	Abim Hospital	Rwekubo HCIV
Soroti RRH	Kagadi Hospital	Sembabule HCIV
	Bwera General Hospital	
	Moyo Hospital	
	Iganga Hospital	
	Rushere Hospital	
	Pallisa Hospital	
	Nebbi General Hospital	
	Atutur Hospital	
	Masindi Hospital	
	Kiboga Hospital	
	Kiryandongo Hospital	
	Kitgum Hospital	
	Bugiri Hospital	
	Bududa Hospital	

## Abbreviations

COPD: Chronic Obstructive Pulmonary Disease
GH: General Hospital
HCIV: Health Centre Level IV
IEC: Information, Education, and Communication materials
MOH: Ministry of Health
NCD: Non-Communicable Disease
NMS: National Medical Stores
RRH: Regional Referral Hospital
SARA: Service Availability and Readiness Assessment
SURE: Securing Ugandans’ Right to Essential Medicines
WHO: World Health Organization
WHO-PEN: WHO Package of Essential Non-communicable Disease Interventions for Primary Health Care in Low-Resource Settings
