# Supplementary material for: Capacity of Ugandan public sector health facilities to prevent and control non-communicable diseases: an assessment based upon WHO-PEN standards
Source: BMC Health Serv Res. 2018 Aug 6;18:606. doi: 10.1186/s12913-018-3426-x (PMC6080524; doi:10.1186/s12913-018-3426-x)
Supplement: Supplementary file 1 — MOH Needs Assessment Tool. Non-Communicable Diseases Needs Assessment Tool used in this study. (DOCX 93 kb) [file 12913_2018_3426_MOESM1_ESM.docx]

**Non-Communicable Diseases Needs Assessment Tool**

**Ministry of Health**

**Uganda**

**July 2013**


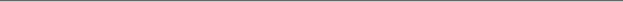


**Background:** Non-communicable diseases (NCDs) are currently the leading killer diseases globally, and are on the rise. About 80% of NCDs deaths occur in low and middle-income countries, including Uganda. The major NCDs in Uganda are diabetes mellitus, hypertension, cardiovascular disease, and some cancers, while others include renal disease, sickle cell disease, and chronic obstructive pulmonary disease (COPD). Among the challenges of NCD control in Uganda are the lack of baseline data on prevalence and risk factors, and the inadequate capacity of the existing health system to provide quality NCDs services.

The Uganda Ministry of Health (MoH) prioritizes NCDs especially in regard to prevention, early diagnosis, and proper management. In partnership with the World Diabetic Foundation (WDF), the MoH has embarked on a countrywide program to build capacity in the control and clinical management of NCDs (with emphasis on diabetes) at the different levels of health care. A Needs Assessment is therefore essential to identify the existing gaps and plan appropriate interventions.

**Purpose:** To assess the capacity of Health Facilities to detect and manage NCDs, as well as identify areas for improvement.

**Overview:** There are **13** main sections of the tool.

They include: **(A)** *Visit Information,* **(B)** *Health Facility Profile*, **(C)** *Human Resource and Skills Profile*, **(D)** *Facility-Based NCD Prevalence*, **(E)** *Clinics and Services*, **(F)** *Equipment*, **(G)** *Medicines & Sundries,* **(H)** *Laboratory*, **(I)** *Costs Related to NCDs,* **(J)** *Referral System,* **(K)** *Health Care Providers’ Skills and Attitudes,* **(L)** *Community Involvement (Engagement)*, and **(M)** *Associations/Patient Groups*.

**Instructions:**

- For a **Yes/No** question (e.g. “Is there a pharmacy in your facility?”), please mark “**YES**” as “**Y**” and **“NO”** as “**N**” in the box provided.
- If the question asks for the “Number” (e.g. “How many weighing scales are there?”), please simply write the number of items in the appropriate box.
- Other questions will show the numbers that correspond to specific answers.
- If the question does not apply, please write “**not applicable**” or “**N/A**”.

**Abbreviations:**

- **NCD:** Non-Communicable Disease
- **DM:** Diabetes Mellitus
- **HT:** Hypertension
- **COPD:** Chronic Obstructive Pulmonary Disease
- **Cardio:** Cardiology
- **SC:** Sickle Cell
- **OB/GY:** Obstetrics/Gynecology
- **MOPD:** Medical Outpatient Department
- **IEC:** Information Education Communication
- **CME:** Continuing Medical Education

**A. VISIT INFORMATION**

|  |  | |  |
| --- | --- | --- | --- |
| A1 | Date of visit (dd/mm/yy) | |  |
| A2 | Officers visiting the Hospital | | 1) |
|  |  |  | 2) |
|  |  |  | 3) |
| A3 | Personnel interviewed^[[1]](#footnote-1)^ | | |
|  | 1 | **Hospital Director:** | |
|  |  | Title/Qualification |  |
|  |  | NCD involvement |  |
|  |  | Contact |  |
|  | 2 | Name |  |
|  |  | Title/Qualification |  |
|  |  | NCD involvement |  |
|  |  | Contact |  |
|  | 3 | Name |  |
|  |  | Title/Qualification |  |
|  |  | NCD involvement |  |
|  |  | Contact |  |
|  | 4 | Name |  |
|  |  | Title/Qualification |  |
|  |  | NCD involvement |  |
|  |  | Contact |  |

**B. HEALTH FACILITY PROFILE**

|  | | | | | | |
| --- | --- | --- | --- | --- | --- | --- |
| B1 | Name of health facility |  | | | | |
| B2 | City/town |  | | | | |
| B3 | Full address of health facility |  | | | | |
| B4 | Is the health facility urban or rural, public or private? | | Urban  Rural | 🞎  🞎 | Public  Private | 🞎  🞎 |
| B5 | Health facility category:  National Referral Hospital – **1**; Regional Referral Hospital – **2**;  General Hospital – **3**; Health Centre IV – **4** | | | | | 🞎 |

**C. HUMAN RESOURCE AND SKILLS PROFILE**

|  | | | | |
| --- | --- | --- | --- | --- |
| **Are the following personnel in the facility, and if so, how many of each specialty/position?** | | | | |
|  | **Specialty/Position** | **Yes/No** | **Number** | **Additional Skills^[[2]](#footnote-2)^** |
| C1 | Cardiologist |  |  |  |
| C2 | Clinical Officer(s) |  |  |  |
| C3 | Community Health Worker(s) |  |  |  |
| C4 | (NCDs) Counsellors |  |  |  |
| C5 | Endocrinologist/Diabetologist |  |  |  |
| C6 | Family physician |  |  |  |
| C7 | Foot care specialist |  |  |  |
| C8 | General surgeon |  |  |  |
| C9 | Laboratory technician(s) |  |  |  |
| C10 | Laboratory technologist(s) |  |  |  |
| C11 | Medical Officer(s) |  |  |  |
| C12 | Medical social workers |  |  |  |
| C13 | Midwives |  |  |  |
| C14 | NCD (DM) Educator |  |  |  |
| C15 | Neurologist |  |  |  |
| C16 | Nurse(s) – general |  |  |  |
| C17 | Nurse(s) – diabetic |  |  |  |
| C18 | Nurse(s) – psychiatric |  |  |  |
| C19 | Nutritionist(s) |  |  |  |
| C20 | Obstetrician/Gynecologist |  |  |  |
| C21 | Oncologist |  |  |  |
| C22 | Ophthalmologist |  |  |  |
| C23 | Paediatrician |  |  |  |
| C24 | Pathologist |  |  |  |
| C25 | Physiotherapist |  |  |  |
| C26 | Psychiatrist |  |  |  |
| C27 | Pulmonologist (or Chest physician) |  |  |  |
| C28 | Radiologist(s) |  |  |  |
| C29 | Radiology technician(s) |  |  |  |
| C30 | Specialist Physician |  |  |  |
| C31 | Vascular Surgeon |  |  |  |
| C32 | Other, specify: |  |  |  |
| **COMMENTS:** | | | | |

**D. FACILITY-BASED NCDs PREVALENCE**

|  | **number of cases IN LAST FINANCIAL YEAR^[[3]](#footnote-3)^** | | **Number** |
| --- | --- | --- | --- |
| D1 | Estimated number of overall patients in your facility^[[4]](#footnote-4)^ | |  |
| D2 | Estimated number of patients with NCDs | |  |
| D3 | Total number of diabetes cases | |  |
| D4 | Adults | |  |
| D5 | Children | |  |
| D6 | Pregnant women | |  |
| D7 | Total number of hypertension cases | |  |
| D8 | Pregnant women | |  |
| D9 | Total number of cancer cases | |  |
| D10 | Breast cancer | |  |
| D11 | Cervix cancer | |  |
| D12 | Prostate cancer | |  |
| D13 | Lung cancer | |  |
| D14 | Liver cancer | |  |
|  | Kaposi’s sarcoma | |  |
| D15 | Other cancer (specify) |  |  |
|  |  |  |  |
|  |  |  |  |
| D16 | Total number of heart disease cases | |  |
| D17 | Adults | |  |
| D18 | Children | |  |
| D19 | Total number of asthma cases | |  |
| D20 | Total number of other COPD cases^[[5]](#footnote-5)^ | |  |
| D21 | Total number of stroke cases | |  |
| D22 | Total number of renal disease cases | |  |
| D23 | Total number of mental health disorder cases | |  |
| D24 | Total number of sickle cell disease cases | |  |
| D25 | Total number of injury cases – road traffic accidents | |  |
| D26 | Total number of injury cases – gender-based violence | |  |
| D27 | Total number of injury cases – trauma due to other causes | |  |
| D28 | Total number of alcohol-related cases | |  |
| D29 | Total number of tobacco-related cases | |  |
| **COMMENTS:** | | | |

**E. CLINICS AND SERVICES**

| **Clinics** | | | | | | | | | | | | | |
| --- | --- | --- | --- | --- | --- | --- | --- | --- | --- | --- | --- | --- | --- |
|  | | **DM** | **HT** | **Cancer** | **Cardio** | **COPD** | **Renal** | **SC** | **OB/GY** | **Paed** | **HIV** | **Surgery** | **MOPD** |
| E1 | Do you have a clinic (NCD)? (**Y/N**) |  |  |  |  |  |  |  |  |  |  |  |  |
| E2 | Do you have a separate (NCD) clinic room? (**Y/N**) |  |  |  |  |  |  |  |  |  |  |  |  |
| E3 | How often is the clinic held? |  |  |  |  |  |  |  |  |  |  |  |  |
| E4 | When is the clinic held? |  |  |  |  |  |  |  |  |  |  |  |  |
| E5 | Is there regular patient review? (**Y/N**) |  |  |  |  |  |  |  |  |  |  |  |  |
| E6 | If yes, how often is each patient reviewed? |  |  |  |  |  |  |  |  |  |  |  |  |
| E7 | Does the clinic conduct performance audits? (**Y/N**) |  |  |  |  |  |  |  |  |  |  |  |  |
| E8 | If yes, specify.^[[6]](#footnote-6)^ |  |  |  |  |  |  |  |  |  |  |  |  |

| **Services** | | | | | | | | | | | | | | | | | | | | | | | | | | | | | | | | | | | |
| --- | --- | --- | --- | --- | --- | --- | --- | --- | --- | --- | --- | --- | --- | --- | --- | --- | --- | --- | --- | --- | --- | --- | --- | --- | --- | --- | --- | --- | --- | --- | --- | --- | --- | --- | --- |
|  |  | | **DM** | **HT** | | **Cancer** | | | **Cardio** | | | **COPD** | | | **Renal** | | | **SC** | | | **OB/GY** | | | **Paed** | | | **HIV** | | | **Surgery** | | | | **MOPD** | |
|  | **Are the following services offered?** | | **Y/N** | **Y/N** | | **Y/N** | | | **Y/N** | | | **Y/N** | | | **Y/N** | | | **Y/N** | | | **Y/N** | | | **Y/N** | | | **Y/N** | | | **Y/N** | | | | **Y/N** | |
| E9 | Blood Pressure measurement | |  |  | |  | | |  | | |  | | |  | | |  | | |  | | |  | | |  | | |  | | | |  | |
| E10 | Weight | |  |  | |  | | |  | | |  | | |  | | |  | | |  | | |  | | |  | | |  | | | |  | |
| E11 | Height | |  |  | |  | | |  | | |  | | |  | | |  | | |  | | |  | | |  | | |  | | | |  | |
| E12 | BMI calculation | |  |  | |  | | |  | | |  | | |  | | |  | | |  | | |  | | |  | | |  | | | |  | |
| E13 | Waist:Hip ratio | |  |  | |  | | |  | | |  | | |  | | |  | | |  | | |  | | |  | | |  | | | |  | |
| E14 | Blood glucose | |  |  | |  | | |  | | |  | | |  | | |  | | |  | | |  | | |  | | |  | | | |  | |
| E15 | Oral Glucose Tolerance Test (OGTT) | |  |  | |  | | |  | | |  | | |  | | |  | | |  | | |  | | |  | | |  | | | |  | |
| E16 | Blood lipids | |  |  | |  | | |  | | |  | | |  | | |  | | |  | | |  | | |  | | |  | | | |  | |
| E17 | Urinalysis | |  |  | |  | | |  | | |  | | |  | | |  | | |  | | |  | | |  | | |  | | | |  | |
| E18 | Urine protein | |  |  | |  | | |  | | |  | | |  | | |  | | |  | | |  | | |  | | |  | | | |  | |
| E19 | Urine ketones | |  |  | |  | | |  | | |  | | |  | | |  | | |  | | |  | | |  | | |  | | | |  | |
| E20 | Eye examination | |  |  | |  | | |  | | |  | | |  | | |  | | |  | | |  | | |  | | |  | | | |  | |
| E21 | Individual patient NCD education | |  |  | |  | | |  | | |  | | |  | | |  | | |  | | |  | | |  | | |  | | | |  | |
| E22 | Group NCD education | |  |  | |  | | |  | | |  | | |  | | |  | | |  | | |  | | |  | | |  | | | |  | |
| E23 | Foot care for Diabetic patients | |  |  | |  | | |  | | |  | | |  | | |  | | |  | | |  | | |  | | |  | | | |  | |
| E24 | Nutrition advice for all patients | |  |  | |  | | |  | | |  | | |  | | |  | | |  | | |  | | |  | | |  | | | |  | |
| E25 | Sickle cell screening (incl. newborns) | |  |  | |  | | |  | | |  | | |  | | |  | | |  | | |  | | |  | | |  | | | |  | |
| E26 | Cervical cancer screening (pap smear) | |  |  | |  | | |  | | |  | | |  | | |  | | |  | | |  | | |  | | |  | | | |  | |
| E27 | Breast cancer screening | |  |  | |  | | |  | | |  | | |  | | |  | | |  | | |  | | |  | | |  | | | |  | |
| E28 | Prostate cancer screening | |  |  | |  | | |  | | |  | | |  | | |  | | |  | | |  | | |  | | |  | | | |  | |
| E29 | Cytology/pathology services | |  |  | |  | | |  | | |  | | |  | | |  | | |  | | |  | | |  | | |  | | | |  | |
| E30 | Palliative care | |  |  | |  | | |  | | |  | | |  | | |  | | |  | | |  | | |  | | |  | | | |  | |
| E31 | Chemotherapy | |  |  | |  | | |  | | |  | | |  | | |  | | |  | | |  | | |  | | |  | | | |  | |
| E32 | Radiotherapy | |  |  | |  | | |  | | |  | | |  | | |  | | |  | | |  | | |  | | |  | | | |  | |
| E33 | Physiotherapy | |  |  | |  | | |  | | |  | | |  | | |  | | |  | | |  | | |  | | |  | | | |  | |
| E34 | Record of family history of NCDs | |  |  | |  | | |  | | |  | | |  | | |  | | |  | | |  | | |  | | |  | | | |  | |
| E35 | NCD patient card | |  |  | |  | | |  | | |  | | |  | | |  | | |  | | |  | | |  | | |  | | | |  | |
| E36 | Provision of NCDs IEC materials | |  |  | |  | | |  | | |  | | |  | | |  | | |  | | |  | | |  | | |  | | | |  | |
| E37 | If you answered yes to #E36, please list examples^[[7]](#footnote-7)^ | | _____________________________________________________________________________________________________ | | | | | | | | | | | | | | | | | | | | | | | | | | | | | | | | |
| E38 | Patient treatment plans | |  | |  | | |  | | |  | | |  | | |  | | |  | | |  | | |  | | |  | | |  | | | |
| E39 | Self-management support^[[8]](#footnote-8)^ | |  | |  | | |  | | |  | | |  | | |  | | |  | | |  | | |  | | |  | | |  | | | |
| E40 | Peer/social support linkage^[[9]](#footnote-9)^ | |  | |  | | |  | | |  | | |  | | |  | | |  | | |  | | |  | | |  | | |  | | | |
| **Guidelines** | | | | | | | | | | | | | | | | | | | | | | | | | | | | | | | | | | | |
|  | |  | **DM** | **HT** | | **Cancer** | | | **Cardio** | | | **COPD** | | | **Renal** | | | **SC** | | | **OB/GY** | | | **Paed** | | | **HIV** | | | **Surg** | | | | **MOPD** |  |
|  | | **Are the following guidelines used?** | **Y/N** | **Y/N** | | | **Y/N** | | | **Y/N** | | | **Y/N** | | | **Y/N** | | | **Y/N** | | | **Y/N** | | | **Y/N** | | | **Y/N** | | | **Y/N** | | **Y/N** | |  |
| E41 | | Diabetes management |  |  | | |  | | |  | | |  | | |  | | |  | | |  | | |  | | |  | | |  | |  | |  |
| E42 | | Hypertension management |  |  | | |  | | |  | | |  | | |  | | |  | | |  | | |  | | |  | | |  | |  | |  |
| E43 | | Hyperlipidemia management |  |  | | |  | | |  | | |  | | |  | | |  | | |  | | |  | | |  | | |  | |  | |  |
| E44 | | Tobacco screening & treatment |  |  | | |  | | |  | | |  | | |  | | |  | | |  | | |  | | |  | | |  | |  | |  |
| E45 | | Alcohol screening & treatment |  |  | | |  | | |  | | |  | | |  | | |  | | |  | | |  | | |  | | |  | |  | |  |
| E46 | | Cancer (cervical, breast, prostate) screening & treatment |  |  | | |  | | |  | | |  | | |  | | |  | | |  | | |  | | |  | | |  | |  | |  |
| E47 | | Mental health screening & treatment |  |  | | |  | | |  | | |  | | |  | | |  | | |  | | |  | | |  | | |  | |  | |  |
| E48 | | Asthma management |  |  | | |  | | |  | | |  | | |  | | |  | | |  | | |  | | |  | | |  | |  | |  |
| E49 | | Sickle cell screening & management |  |  | | |  | | |  | | |  | | |  | | |  | | |  | | |  | | |  | | |  | |  | |  |
| E50 | | Palliative care |  |  | | |  | | |  | | |  | | |  | | |  | | |  | | |  | | |  | | |  | |  | |  |
| **COMMENTS:** (include type of guidelines used, e.g. WHO, UCG – Uganda Clinical Guidelines or IDF – International Diabetic Federation). Also mention about any Rehabilitation services. | | | | | | | | | | | | | | | | | | | | | | | | | | | | | | | | | | | |

| **HIV clinic** | | |
| --- | --- | --- |
| **Number of known HIV patients** | | |
| E39 | Total number of active patients with HIV in the last financial year^[[10]](#footnote-10)^ | Male:  Female: |
| E40 | Number of new cases in last 3 financial years | 2012-13: |
|  |  | 2011-12: |
|  |  | 2010-11: |
| E41 | Number of active HIV patients with cancer |  |
| E42 | Number of active HIV patients with diabetes |  |
| E43 | Number of active HIV patients with hypertension |  |
| E44 | Number of active HIV patients with CVD |  |
| E45 | Number of active HIV patients with liver disease |  |
| E46 | Number of active HIV patients with kidney disease |  |
| **COMMENTS:** | | |

**F. EQUIPMENT**

|  | | | | | **DM** | | **HT** | | **Cancer** | | **Cardio** | | **COPD** | | **Renal** | | **SC** | | | **OB/GY** | | **Paed** | | **HIV** | | **Surgery** | | **MOPD** | |
| --- | --- | --- | --- | --- | --- | --- | --- | --- | --- | --- | --- | --- | --- | --- | --- | --- | --- | --- | --- | --- | --- | --- | --- | --- | --- | --- | --- | --- | --- |
| **Is the following equipment available in the NCD clinic? (Y/N and number)** | | | | | **Y/N** | **#** | **Y/N** | **#** | **Y/N** | **#** | **Y/N** | **#** | **Y/N** | **#** | **Y/N** | **#** | **Y/N** | | **#** | **Y/N** | **#** | **Y/N** | **#** | **Y/N** | **#** | **Y/N** | **#** | **Y/N** | **#** |
| F1 | Clinicians table | | | |  |  |  |  |  |  |  |  |  |  |  |  |  | |  |  |  |  |  |  |  |  |  |  |  |
| F2 | Nurses station/table | | | |  |  |  |  |  |  |  |  |  |  |  |  |  | |  |  |  |  |  |  |  |  |  |  |  |
| F3 | Hand washing basin/sink with soap | | | |  |  |  |  |  |  |  |  |  |  |  |  |  | |  |  |  |  |  |  |  |  |  |  |  |
| F4 | Patient files | | | |  |  |  |  |  |  |  |  |  |  |  |  |  | |  |  |  |  |  |  |  |  |  |  |  |
| F5 | File cabinet/cupboard/storage space | | | |  |  |  |  |  |  |  |  |  |  |  |  |  | |  |  |  |  |  |  |  |  |  |  |  |
| F6 | NCD register: for new cases only | | | |  | |  | |  | |  | |  | |  | |  | | |  | |  | |  | |  | |  | |
| F7 | NCD register: for follow-up cases only | | | |  | |  | |  | |  | |  | |  | |  | | |  | |  | |  | |  | |  | |
| F8 | NCD register: for admission only | | | |  | |  | |  | |  | |  | |  | |  | | |  | |  | |  | |  | |  | |
| F9 | BP machine: Mercury sphygmoman-ometer | | | Number present |  | |  | |  | |  | |  | |  | |  | | |  | |  | |  | |  | |  | |
| F10 |  |  |  | Number functional |  | |  | |  | |  | |  | |  | |  | | |  | |  | |  | |  | |  | |
| F11 |  |  |  | # Calibrated |  | |  | |  | |  | |  | |  | |  | | |  | |  | |  | |  | |  | |
| F12 | BP machine: Aneroid | | | Number present |  | |  | |  | |  | |  | |  | |  | | |  | |  | |  | |  | |  | |
| F13 |  |  |  | Number functional |  | |  | |  | |  | |  | |  | |  | | |  | |  | |  | |  | |  | |
| F14 |  |  |  | # Calibrated |  | |  | |  | |  | |  | |  | |  | | |  | |  | |  | |  | |  | |
| F15 | BP machine: Automated | | | Number present |  | |  | |  | |  | |  | |  | |  | | |  | |  | |  | |  | |  | |
| F16 |  |  |  | Number functional |  | |  | |  | |  | |  | |  | |  | | |  | |  | |  | |  | |  | |
| F17 |  |  |  | # Calibrated |  | |  | |  | |  | |  | |  | |  | | |  | |  | |  | |  | |  | |
| F18 | BP cuffs: Standard (25 cm x 12 cm) | | | |  | |  | |  | |  | |  | |  | |  | | |  | |  | |  | |  | |  | |
| F19 | BP cuffs: Alternate (36 cm x 12 cm) | | | |  | |  | |  | |  | |  | |  | |  | | |  | |  | |  | |  | |  | |
| F20 | BP cuffs: Paediatric | | | |  | |  | |  | |  | |  | |  | |  | | |  | |  | |  | |  | |  | |
| F21 | Stethoscope | | Number present | |  | |  | |  | |  | |  | |  | |  | | |  | |  | |  | |  | |  | |
| F22 |  |  | Number functional | |  | |  | |  | |  | |  | |  | |  | | |  | |  | |  | |  | |  | |
| F23 | Blood  Glucose meter | | Number present | |  | |  | |  | |  | |  | |  | |  | | |  | |  | |  | |  | |  | |
| F24 |  |  | Number functional | |  | |  | |  | |  | |  | |  | |  | | |  | |  | |  | |  | |  | |
| F25 |  |  | # Calibrated correctly | |  | |  | |  | |  | |  | |  | |  | | |  | |  | |  | |  | |  | |
| F26 |  |  | Cost of strips per unit | |  | |  | |  | |  | |  | |  | |  | | |  | |  | |  | |  | |  | |
| F27 | Urine testing strips | | Multiple test | |  | |  | |  | |  | |  | |  | |  | | |  | |  | |  | |  | |  | |
| F29 |  |  | Availability? Always – **1**; Sometimes – **2**; Never – **0** | |  | |  | |  | |  | |  | |  | |  | | |  | |  | |  | |  | |  | |
| F30 | Weighing scales | | # Bathroom type | |  | |  | |  | |  | |  | |  | |  | | |  | |  | |  | |  | |  | |
| F31 |  |  | # Hospital type | |  | |  | |  | |  | |  | |  | |  | | |  | |  | |  | |  | |  | |
| F32 | Height meters | | Number present | |  | |  | |  | |  | |  | |  | |  | | |  | |  | |  | |  | |  | |
| F33 |  |  | # Calibrated correctly | |  | |  | |  | |  | |  | |  | |  | | |  | |  | |  | |  | |  | |
| F34 | Ophthalmo-scope | | Number present | |  | |  | |  | |  | |  | |  | |  | | |  | |  | |  | |  | |  | |
| F35 |  |  | Number functional | |  | |  | |  | |  | |  | |  | |  | | |  | |  | |  | |  | |  | |
| F36 | Snellen charts (visual acuity exam) | | # Handheld type | |  | |  | |  | |  | |  | |  | |  | | |  | |  | |  | |  | |  | |
| F37 |  |  | # Distance type | |  | |  | |  | |  | |  | |  | |  | | |  | |  | |  | |  | |  | |
|  | | | | | **DM** | | **HT** | | **Cancer** | | **Cardio** | | **COPD** | | **Renal** | | **SC** | | | **OB/GY** | | **Paed** | | **HIV** | | **Surgery** | | **MOPD** | |
|  | **Other equipment** | | | | **Y/N** | **#** | **Y/N** | **#** | **Y/N** | **#** | **Y/N** | **#** | **Y/N** | **#** | **Y/N** | **#** | **Y/N** | | **#** | **Y/N** | **#** | **Y/N** | **#** | **Y/N** | **#** | **Y/N** | **#** | **Y/N** | **#** |
| F38 | BMI chart | | | |  |  |  |  |  |  |  |  |  |  |  |  |  | |  |  |  |  |  |  |  |  |  |  |  |
| F39 | Measuring tapes | | | |  |  |  |  |  |  |  |  |  |  |  |  |  | |  |  |  |  |  |  |  |  |  |  |  |
| F40 | Tuning forks | | | |  |  |  |  |  |  |  |  |  |  |  |  |  | |  |  |  |  |  |  |  |  |  |  |  |
| F41 | Monofilament | | | |  |  |  |  |  |  |  |  |  |  |  |  |  | |  |  |  |  |  |  |  |  |  |  |  |
| F42 | Spirometer | | | |  |  |  |  |  |  |  |  |  |  |  |  |  | |  |  |  |  |  |  |  |  |  |  |  |
| F43 | Spacers for inhalers | | | |  |  |  |  |  |  |  |  |  |  |  |  |  | |  |  |  |  |  |  |  |  |  |  |  |
| F44 | Nebuliser | | | |  |  |  |  |  |  |  |  |  |  |  |  |  | |  |  |  |  |  |  |  |  |  |  |  |
| F45 | Thermometers | | | |  |  |  |  |  |  |  |  |  |  |  |  |  | |  |  |  |  |  |  |  |  |  |  |  |
| F46 | Speculum (cervix) | | | |  |  |  |  |  |  |  |  |  |  |  |  |  | |  |  |  |  |  |  |  |  |  |  |  |
| F47 | Bronchoscope | | | |  |  |  |  |  |  |  |  |  |  |  |  |  | |  |  |  |  |  |  |  |  |  |  |  |
| F48 | Proctoscope | | | |  |  |  |  |  |  |  |  |  |  |  |  |  | |  |  |  |  |  |  |  |  |  |  |  |
| F49 | Colonoscope | | | |  |  |  |  |  |  |  |  |  |  |  |  |  | |  |  |  |  |  |  |  |  |  |  |  |
| F50 | Autoscope | | | |  |  |  |  |  |  |  |  |  |  |  |  |  | |  |  |  |  |  |  |  |  |  |  |  |
| F51 | Emergency Trolley/Tray^[[11]](#footnote-11)^ | | | |  |  |  |  |  |  |  |  |  |  |  |  |  | |  |  |  |  |  |  |  |  |  |  |  |
| F52 | Physiotherapy equipment | | | |  |  |  |  |  |  |  |  |  |  |  |  |  | |  |  |  |  |  |  |  |  |  |  |  |
| F53 | Other  (Specify) |  | | |  |  |  |  |  |  |  |  |  |  |  |  |  | |  |  |  |  |  |  |  |  |  |  |  |
|  |  |  | | |  |  |  |  |  |  |  |  |  |  |  |  |  | |  |  |  |  |  |  |  |  |  |  |  |
|  |  |  | | |  |  |  |  |  |  |  |  |  |  |  |  |  | |  |  |  |  |  |  |  |  |  |  |  |
|  | **Maintenance** | | | | **Y/N** | | **Y/N** | | **Y/N** | | **Y/N** | | **Y/N** | | **Y/N** | | **Y/N** | | | **Y/N** | | **Y/N** | | **Y/N** | | **Y/N** | | **Y/N** | |
| F54 | Is there a written equipment maintenance plan? | | | |  | |  | |  | |  | |  | |  | |  | | |  | |  | |  | |  | |  | |
| F55 | If yes, is the plan implemented? | | | |  | |  | |  | |  | |  | |  | |  | | |  | |  | |  | |  | |  | |
|  | **Imaging** | | | | **Available?** | | | | | | | | | | | | | **Functional?** | | | | | | | | | | | |
| F56 | Ultrasound scan | | | |  | | | | | | | | | | | | |  | | | | | | | | | | | |
| F57 | Echography | | | |  | | | | | | | | | | | | |  | | | | | | | | | | | |
| F58 | ECG monitor | | | |  | | | | | | | | | | | | |  | | | | | | | | | | | |
| F59 | X-Rays | | | |  | | | | | | | | | | | | |  | | | | | | | | | | | |
| F60 | Doppler | | | |  | | | | | | | | | | | | |  | | | | | | | | | | | |
| F61 | CT-Scan | | | |  | | | | | | | | | | | | |  | | | | | | | | | | | |
| F62 | Other, specify | | | |  | | | | | | | | | | | | |  | | | | | | | | | | | |
|  | **Power Supply** | | | | **Available?** | | | | | | | | | | | | **Functional?** | | | | | | | | | | | | |
| F63 | Reliable power supply | | | |  | | | | | | | | | | | |  | | | | | | | | | | | | |
| F64 | Alternative source of power^[[12]](#footnote-12)^ | | | |  | | | | | | | | | | | |  | | | | | | | | | | | | |
| **COMMENTS:**  **________________________________________________________________________________________________________________________________________________________________________________________________________________________________________________________________________________________________________________________________________________________________________________________________________________________________________________________________________________________________________________________________________________________________________________________________________________________________________________________________________________________________________________________________________________________________________________________________________________________________________________________________________________________________________________________________________________________________________________________________________________________________________________________________________________________________________________________________________________________________________________________________________**  **_________________________________________________________________________________________________________________________________________________**  **_________________________________________________________________________________________________________________________________________________** | | | | | | | | | | | | | | | | | | | | | | | | | | | | | |

**G. MEDICINES AND SUNDRIES**

| G1 | Is there a drug store (pharmacy) in your facility? (**Y/N**) | | | | |  | | |
| --- | --- | --- | --- | --- | --- | --- | --- | --- |
| **Medicines** | | | | | | | | |
|  | **Classes of drugs** | | **Available** | **How often are they stocked?** | | | **Was there a stock-out in the last quarter?** | **Was there a stock-out in the last financial year?** |
|  |  |  |  | **Every 2 mos.**  **Quarterly**  **Other** | **1**  **23** | |  |  |
|  | Anti-hypertensives | |  |  | | |  |  |
| G2 | Thiazide diuretic (e.g. Aprinox) | |  |  | | |  |  |
| G3 | Calcium channel blocker (e.g. Nifedipine) | |  |  | | |  |  |
| G4 | Beta-blocker (e.g. Propranolol) | |  |  | | |  |  |
| G5 | ACE inhibitor (e.g. Captopril) | |  |  | | |  |  |
| G6 | Others (e.g. Aldomet, Hydrallazine, Magnesium Sulphate) | |  |  | | |  |  |
|  | Diabetic drugs | |  |  | | |  |  |
| G7 | Biguanides (e.g. Metformin) | |  |  | | |  |  |
| G8 | Sulfonylureas (e.g. Glibenclamide) | |  |  | | |  |  |
| G9 | Thiazolidinediones (e.g. Pioglitazone) | |  |  | | |  |  |
| G10 | Dipeptidyl peptidase-4 inhibitors | |  |  | | |  |  |
| G11 | Alpha-glucosidase inhibitors | |  |  | | |  |  |
| G12 | Others | |  |  | | |  |  |
| G13 | Insulin type available | Ultra short-acting |  |  | | |  |  |
| G14 |  | Short-acting |  |  | | |  |  |
| G15 |  | Intermediate |  |  | | |  |  |
| G16 |  | Long-acting |  |  | | |  |  |
| G17 | Strength of insulin available | U 100 |  |  | | |  |  |
|  |  | Other |  |  | | |  |  |
| G18 | Insulin syringes (e.g. U100) | |  |  | | |  |  |
|  | For other diseases | |  |  | | |  |  |
| G19 | Folic acid | |  |  | | |  |  |
| G20 | Statins | |  |  | | |  |  |
| G21 | Cardiac Aspirin | |  |  | | |  |  |
| G22 | Sulfadoxine/pyrimethamine (e.g. Fansidar) | |  |  | | |  |  |
| G23 | Antibiotics, specify: | |  |  | | |  |  |
| G24 | Anticoagulants | |  |  | | |  |  |
| G25 | Anticancer, specify: | |  |  | | |  |  |
| G26 | Other^[[13]](#footnote-13)^ | |  |  | | |  |  |
| **Other Questions** | | | | | | | | |
|  | What is the source of procurement? | | | | | | | |
| G27 | Government | | | | |  | | |
| G28 | Other, and specify^[[14]](#footnote-14)^ | | | | |  | | |
| G29 | Do you get the types of drugs you ask for? | | | | |  | | |
| G30 | Do you get the quantity of drugs you ask for? | | | | |  | | |
| G31 | Do you have a refrigerator in the drug store (pharmacy)? | | | | |  | | |
| **COMMENTS:** | | | | | | | | |

**H. LABORATORY**

|  | **Question** | **Yes/NO** |
| --- | --- | --- |
| H1 | Is there a laboratory in the facility? |  |
| Are the following lab tests done? | | |
| H2 | Bacteriology including culture and sensitivity |  |
| H3 | Blood Grouping and Cross-match |  |
| H4 | Cytology |  |
| H5 | Electrolytes (e.g., potassium) |  |
| H6 | Full blood count and differential |  |
| H7 | Full Urinalysis |  |
| H8 | Hb electrophoresis |  |
| H9 | HbA1c |  |
| H10 | Hemoccult |  |
| H11 | Hemoglobin |  |
| H12 | Hormonal Assays (other) |  |
| H13 | Lipid Profile |  |
| H14 | Liver Function Tests |  |
| H15 | Microalbuminuria |  |
| H16 | Pregnancy test |  |
| H17 | PSA |  |
| H18 | Random Blood Sugar |  |
| H19 | Renal Function Tests |  |
| H20 | Sickling test |  |
| H21 | Thick Film |  |
| H22 | Thin Film |  |
| H23 | Thyroid function tests |  |
| H24 | Other, specify^[[15]](#footnote-15)^ |  |
| H25 | Is there a functional centrifuge available? |  |
| H26 | Is there a functional microscope available? |  |
| H27 | Is there a functional refrigerator available? |  |
| **COMMENTS:** | | |

1. **COSTS RELATED TO NCDS^[[16]](#footnote-16)^**

|  | **General Item** | **Specify Item** | **Cost Estimate^[[17]](#footnote-17)^** |
| --- | --- | --- | --- |
| I1 | Drugs |  |  |
|  |  |  |  |
| I2 | Labs |  |  |
|  |  |  |  |
| I3 | Imaging |  |  |
|  |  |  |  |
| I4 | Other Services |  |  |
|  |  |  |  |
| **COMMENTS:** | | | |

**J. REFERRAL SYSTEM**

|  | **question** | | | **yes/no** |
| --- | --- | --- | --- | --- |
| J1 | **Health System:** Availability of protocols for management and referral of NCD patients in this facility. | | |  |
| J2 | **If yes specify:** |  | | |
|  |  |  | | |
|  |  |  | | |
| J3 | **Health System:** Availability of a referral form (or any other form of communication) to the next facility | | Outgoing |  |
|  |  |  | Receiving |  |
| J4 | **Health System:** Availability of transport to the next facility | | |  |
| J5 | **Referral Practicalities:** A register exists to monitor follow-up and gather statistics on referrals | | Outgoing |  |
|  |  |  | Receiving |  |
| J6 | **Supervision and Capacity Building:** The facility has a program that integrates NCDs support supervision and capacity building in lower health facilities | | |  |
| **COMMENTS:** | | | | |

**K. HEALTH CARE PROVIDERS’ SKILLS AND ATTITUDES**

| **Access** | | | | | | | |
| --- | --- | --- | --- | --- | --- | --- | --- |
|  | Do you have access to the following resources? | | | | Yes/No | | |
| K1 | Facility-specific NCD guidelines | | | |  | | |
| K2 | National/WHO NCD guidelines | | | |  | | |
| K3 | NCD journal articles | | | |  | | |
| K4 | Do you receive “medical alerts”^[[18]](#footnote-18)^ on NCD patients? | | | |  | | |
| K5 | Do you receive NCD support supervision? | | | |  | | |
| **SKILLS (target NCDs team)** | | | | | | | |
|  | | **Phys.** | **MOs** | **Cos** | | **Nurse** | **Other** |
| K6 | How many have received any special training or CME on NCDs? |  |  |  | |  |  |
|  | How many have received training or CME in the following areas? | | | | | | |
| K7 | Prevention/management of hypertension |  |  |  | |  |  |
| K8 | Prevention/management of diabetes |  |  |  | |  |  |
| K9 | Prevention/management of heart disease |  |  |  | |  |  |
| K10 | Screening breast cancer |  |  |  | |  |  |
| K11 | Screening cervical cancer |  |  |  | |  |  |
| K12 | Diagnosis/management of asthma |  |  |  | |  |  |
| K13 | Diagnosis/management of COPD |  |  |  | |  |  |
| K14 | Screening/diagnosis/management of sickle cell disease |  |  |  | |  |  |
| K15 | Screening/diagnosis/management of depression & mental health d/o |  |  |  | |  |  |
| K16 | Screening/management tobacco abuse |  |  |  | |  |  |
| K17 | Screening/management alcohol abuse |  |  |  | |  |  |
| K18 | Palliative care |  |  |  | |  |  |
| **COMMENTS:** | | | | | | | |

**DO NOT COMPLETE PAGE 16 AT THIS TIME**

**Individual Provider Survey**

| K18 | **Cadre:** |  | |
| --- | --- | --- | --- |
| K19 | **Clinic:** |  | |
|  | **CONFIDENCE IN NCD MANAGEMENT** | |  |
|  | Not at all – **1**; Somewhat – **2**; Confident – **3**; Very confident - **4** | | Response |
| K20 | Hypertension | |  |
| K21 | Diabetes | |  |
| K22 | High cholesterol | |  |
| K23 | Screening for cervical cancer | |  |
| K24 | Screening for breast cancer | |  |
| K25 | Diagnosis/management of asthma | |  |
| K26 | Screening/management of depression & other mental health disorders | |  |
| K27 | Screening/diagnosis/management of sickle cell disease | |  |
| K28 | Treatment of tobacco abuse | |  |
| K29 | Treatment of alcohol abuse | |  |
| **ATTITUDES** | | | |
|  | Agree – **1**; Disagree – **2**; No opinion – **3** | | Response |
| K30 | There are no effective depression treatments that can be provided by a primary care physician in my facility. | |  |
| K31 | My clinical training prepared me adequately to manage chronic diseases. | |  |
| K32 | A doctor can influence whether a patient successfully quits smoking. | |  |
| K33 | I am able to spend the time I need to provide good medical care for my patients with chronic diseases. | |  |
| K34 | Patients receive better care for chronic medical conditions if they have a designated primary care provider. | |  |
| K35 | My facility has the capacity to manage chronic diseases. | |  |

**DO NOT COMPLETE PAGE 16 AT THIS TIMEL. COMMUNITY INVOLVEMENT (ENGAGEMENT)**

|  |  | **y/n** |
| --- | --- | --- |
| L1 | Health facility has formal linkages to community – regular meetings with community on NCDS |  |
| L2 | Health facility communicates with community about disease states through media, gatherings, IEC, and/or village health teams |  |
| L3 | Health facility participates in partnerships with community organizations that focus on NCDs |  |
| L4 | Health facility engages community in patient follow-up |  |
| **COMMENTS:** | | |

**M. ASSOCIATIONS/PATIENT GROUPS**

| **uganda diabetic association** | | | |
| --- | --- | --- | --- |
| M1 | Do you have a branch of UDA in your facility? (**Y/N**) | |  |
| M2 | Is the branch functional?^[[19]](#footnote-19)^ |  | |
| M3 | Contact information for your UDA branch | Contact person:  Address:  Telephone:  Email: | |
| M4 | What do you need from UDA head office? |  | |
| M5 | If no, are you willing to open a UDA branch at your facility? (**Y/N**) | |  |
| M6 | Contact information for person interested in heading branch | Contact person:  Title:  Telephone:  Email: | |
| **OTHER ASSOCIATIONS/GROUPS** | | | |
|  | **Association/Group** | **Contact information** | |
| M7 |  |  | |
| M8 |  |  | |
| M9 |  |  | |
| **COMMENTS:** | | | |

1. Personnel interviewed should be from any of the following areas: Administration, Pharmacy, Clinicians in NCD clinics (including nurses), Records, and/or Laboratory. [↑](#footnote-ref-1)
2. e.g. Foot care, palliative care, nutrition, cancer screening, sickle cell screening, mental health skills, and any other skill. Please list skill and then number of personnel with the specified skill. For example: Foot care (2). [↑](#footnote-ref-2)
3. Please obtain actual number of patients from register. [↑](#footnote-ref-3)
4. Both inpatient (admission) and outpatient. [↑](#footnote-ref-4)
5. COPD – defined as progressive symptoms of cough, and/or sputum production, and/or dyspnea for more than 3 months where asthma and infectious causes have been excluded. [↑](#footnote-ref-5)
6. e.g. loss to follow-up, deaths, availability of drugs, etc. [↑](#footnote-ref-6)
7. Brochures, booklets, magazines, CDs, dummies, posters, audiotapes, etc. [↑](#footnote-ref-7)
8. e.g. Lifestyle modification [↑](#footnote-ref-8)
9. Including expert patients [↑](#footnote-ref-9)
10. An “active patient” has attended the HIV clinic within the last 6 months. [↑](#footnote-ref-10)
11. Please specify under comments what your emergency trolley/tray contains. [↑](#footnote-ref-11)
12. e.g. standby generator, solar, etc. [↑](#footnote-ref-12)
13. Include renal, and other drugs. [↑](#footnote-ref-13)
14. e.g. Special fund for health, independent wholesaler, gift from a philanthropic body [↑](#footnote-ref-14)
15. e.g. Carcinoembryonic Antigen [↑](#footnote-ref-15)
16. Costs for patients related to NCDs. [↑](#footnote-ref-16)
17. Estimate cost for 1 month’s supply of drugs and 1 visit for labs, imaging, and other services. [↑](#footnote-ref-17)
18. “Medical alerts” refers to any form of identification for NCDs patients e.g. Health cards, sticker on patient’s file, etc. [↑](#footnote-ref-18)
19. e.g. Regular meetings held, minutes available, work plans available, reports available and/or submitted to UDA head office, etc. [↑](#footnote-ref-19)
